# A Joint Resource Allocation, Security with Efficient Task Scheduling in Cloud Computing Using Hybrid Machine Learning Techniques

**DOI:** 10.3390/s22031242

**Published:** 2022-02-06

**Authors:** Prasanta Kumar Bal, Sudhir Kumar Mohapatra, Tapan Kumar Das, Kathiravan Srinivasan, Yuh-Chung Hu

**Affiliations:** 1 Department of Computer Science and Engineering, GITA Autonomous College, Bhubaneswar 751012, India; prasantakbal9@gmail.com; 2 Faculty of Emerging Technologies, Sri Sri University, Cuttack 754006, India; sudhir.mohapatra@srisriuniversity.edu.in; 3 School of Information Technology and Engineering, Vellore Institute of Technology, Vellore 632014, India; tapan.das@vit.ac.in; 4 School of Computer Science and Engineering, Vellore Institute of Technology, Vellore 632014, India; kathiravan.srinivasan@vit.ac.in; 5 Department of Mechanical and Electromechanical Engineering, National Ilan University, Yilan 26047, Taiwan

**Keywords:** cloud computing, resource allocation, task scheduling, data storage, cloud security, hybrid machine learning, RATS-HM, NSUPREME

## Abstract

The rapid growth of cloud computing environment with many clients ranging from personal users to big corporate or business houses has become a challenge for cloud organizations to handle the massive volume of data and various resources in the cloud. Inefficient management of resources can degrade the performance of cloud computing. Therefore, resources must be evenly allocated to different stakeholders without compromising the organization’s profit as well as users’ satisfaction. A customer’s request cannot be withheld indefinitely just because the fundamental resources are not free on the board. In this paper, a combined resource allocation security with efficient task scheduling in cloud computing using a hybrid machine learning (RATS-HM) technique is proposed to overcome those problems. The proposed RATS-HM techniques are given as follows: First, an improved cat swarm optimization algorithm-based short scheduler for task scheduling (ICS-TS) minimizes the make-span time and maximizes throughput. Second, a group optimization-based deep neural network (GO-DNN) for efficient resource allocation using different design constraints includes bandwidth and resource load. Third, a lightweight authentication scheme, i.e., NSUPREME is proposed for data encryption to provide security to data storage. Finally, the proposed RATS-HM technique is simulated with a different simulation setup, and the results are compared with state-of-art techniques to prove the effectiveness. The results regarding resource utilization, energy consumption, response time, etc., show that the proposed technique is superior to the existing one.

## 1. Introduction

Cloud computing is a remarkable innovation created by the forefront season of labourer farms in PC, and it helps in discontinuing virtualization movements [1]. Appropriate handling is portrayed as an “association” that wires programming, foundation as a help, and Platform as a Service (PaaS) [2,3]. Everybody has a different all-around proposition about business. The aim of appropriated figuring is to create a virtual asset of PCs, workers, and specialists develop that application in order to serve the clients, paying little mind to the procured model [4]. In addition, Internet connectivity and infrastructure are important since the cloud is built on the bedrock of two major foundations, e.g., cloud computing and networking. For many cloud applications, the network can be used for cloud computing and additional applications [5]. The QoS distribution network in the cloud is integrated with its infrastructure and capabilities. As a result, more application service providers (ASPs) [6] realize the distinction between the actual use and operation of the required infrastructure and have used the infrastructure leased from the infrastructure providers. For example, Force Square uses Amazon EC2 Analytics for over 5 million days, saving 53% of its value to meet measurable needs [7], creating the first cloud resource measurement resource.

In particular, pertaining to the demand forecast schedule, the ASP periodically reviews the rental services, makes appropriate decisions about the allocation of goals and resources, and does not spend money on extra calculation, storage, or data transfer [8,9]. Moreover, resource allocation should be commensurate with decentralization, as the provider may deliver services of unique types and or combinations of these services based on resources, complicating the problem rather than requiring complex tenders [10]. Relevant resources can be accessed from multiple sources, and multiple users can compete with the same resources [11,12], with suppliers demanding suppliers, customer submissions, and customer submissions. In cloud computing, most issues relate to data security, power, security, service availability, memory expansion management, and task planning. However, task planning is usually a major topic of cloud computing research. Many tasks in cloud computing require high performance, optimal completion time, low response time, and available resources to utilize useful resources. On account of these varied purposes of the allocation plan, it must assign the tasks correctly.

It can provide services to clients over the Internet using various resources [13]. Since Amazon introduced the cloud computing concept, Amazon has developed several cloud computing systems, including EC2, Google of Engine, Apache Hardtop, and Microsoft Azure. Amazon EC2 is a resource system cluster. Web services are provided by a Linux virtual machine resource, Amazon Data Center [14]. Events can be divided into three categories according to their size: small, large, and very large [15]. There is an influential impact of cloud computing on the IT industry [16] and narrow competition among companies regarding the efficiency of their service delivery [17].

Companies are also striving to improve or upgrade their services further through various resources so that more and more clients can subscribe to the cloud [18]. Hence, one of the most important factors that affect the quality of service is resource allocation and SLA [19], showing the level of user satisfaction. However, dimensions and boundaries must be specified, and upper parameters are difficult to achieve [20].

This study has the following significant contributions:

- In order to manage resource crunches in the cloud environment, we proposed scheduling user tasks by employing the advanced Cat optimization algorithm.
- The proposed resource allocation and security with efficient computer operational planning use hybrid machine learning to optimize the task.
- ICS-TS is introduced to improve passive resources by partitioning the cloud environment into the workspace and state space. GO-DNN based resource management further reduces resource usage in a large-scale cloud environment, with multiple servers receiving multiple requests per day from users.
- On successfully completing the system, an in-depth neural network based on optimization is implemented, setting tasks on appropriate virtual machines. Consequently, the source forecast and reset forecast measures virtual machine processors, memory, and I/O usage.

The rest of the paper is organized as follows: Section 2 discusses about previous work; Section 3 formulates the problem and network model; the proposed research is exhibited in Section 4, Section 5 reveals experimental result, and the paper is concluded in Section 6.

## 2. Related Work

Wei et al. [21] have presented the asset distribution model dependent on distinct asset valuing many SPs and various asset allotments simultaneously, which improves the benefit. The recreation results show that the assessed cost of CSAMIISG is near the genuine exchange cost, and the exchange cost is not exactly the real exchange esteem. The method is comparative for SPs and INs. They will refresh the application framework for future activities and change the settings to make it more effective.

Tang et al. [22] have proposed a YARN’s endeavors to determine these issues. The dispersion of progressive assets is considered at one level. For onetime asset distribution, another asset assignment framework is called long-haul asset reasonableness (LTRF) for such assessments. They offer various leveled long-haul asset reasonableness (H-LTRF) with the option of the LTRF expansion to add progressive sources, for example, the LTRF and H-LTRF. LTYARN subject by presenting LTRF and H-LTRF, and their examinations show that this prompts preferable legitimization of assets over the current assessor.

Zhang et al. [23] have presented a distributed computing that offers asset designation and estimating and offers a practical sale dependent on client evaluations and qualities. Contingent upon the installment model, clients can present many solicitations simultaneously. However, they can deal with different solicitations, one of which is known as an unclear presentation. They show that asset suppliers can receive expanded social rewards and genuine help for the association. They offer a way to deal with asset designation to make brisk assignment arrangements and upgrade the social advantages of cloud asset suppliers. The installment technique quantifies the interests of the asset supplier for every client. They break down the arrangement on a preliminary premise dependent on social help, execution time, asset use, and clients.

Jiang et al. [24] have proposed that the VM joining asset allotment calculation was used to accomplish energy productivity and diminish server farm administration level understanding infringement, considering the utilization of DCNS fragments, the number of overhauls, and the length of the transportation course. This technique effectively decreases energy utilization, the number of movements, and the length of the relocation way to the unique cloud administration.

Gong et al. [25] have presented a resource task control approach that targets dynamic excess weight and resource requirements. It allows various sources to respond to various instabilities following various obstacles and adds consolidated help to join to ensure that QoS does not offer acceptable assistance for one assistance. As showed by close tests, resource use can be improved by giving agreeable sponsoring to resources, regardless of whether there is a specific method to manage organization needs. This system ensures that QoS adjusts to normal mediations and responds continuously to eccentric resource necessities.

Wu et al. [26] have presented a trade CPU and memory hotspots for new programming and VMs with a two-venture crossbreed variation model to expect VM load-dependent on severe order control. Rather than anticipating course events, they considered order line programs using natural language preparing (NLP) innovation and used grey research analysis (GRA) to lessen credits. Built-up a double mixture versatile model that productively and precisely predicts VM load, including CPU and memory. Select projects that expand the CPU VM by over 5%, and afterward assess the ANFIS model VM and CPU and memory load using the Boeing technique. Broad testing shows that rearrangement techniques improved the execution and asset usage of VMs.

Zhao et al. [27] have described an integrated approach based on MEC and Cloud Computing loads for vehicles on the transport network. Cloud-MEC system integrated download results are designed to synchronize system upload results and allow system resources. If the problem worsens and the NPP becomes complicated, they recommend downloading the resource optimization program calculation and switching to the CCORAO program associated with the solution. The algorithm effectively improves computer usage and computation time, especially if MEC servers do not meet the requirements because of insufficient computing resources.

Abbasi et al. [28] have proposed power consumption to balance power consumption and load delay, i.e., XCS and BCM-XCS. The results of our experiments show the advantages of BCM-XCS compared to the basic method based on XCS. Load distribution between cloud and fog nodes is a specific way of reducing processing delays and communication delays. The major advantage of controlling processing fluctuations is that specific methods can gradually reduce processing delays by 42%, using specific energy consumption.

Reis et al. [29] have investigated a predictive approach to configuration recommendation based on genetic algorithms (GA) and support vector regression (SVR). This integrated program calculates idle time and provides possible and optimal configuration of cloud resources in terms of time and cost. The results showed that the forecast time was very close to direct time, which effectively estimated time and costs and their reduction.

Gui et al. [30] have proposed tests on load and resource distribution on a dense C-Ron operating MEC designed to improve single-phase energy efficiency. A standard hybrid non-software program designed to improve load unloading results, resource planning, and radio resource allocation. Lebanon has developed a theory that divides the problem into four individual sub-problems using central variation methods and compatibility games. They theoretically analyze the trade-off between service delays and energy efficiency. Advanced simulations exhibit how system parameters influence energy efficiency and service interruption. The results of the models confirmed the benefits of a rich C-RON load and resource allocation scheme.

Praveenchandar et al. [31] recommended an energy-saving approach for effective planning and resource allocation. Resource allocation in task execution and response time was fulfilled using the forecasting system and dynamic resource updating algorithm. This system is useful for reducing the energy structure of the system by reducing data center consumption. The resource table update approach returns the exact values. Resource allocation is effectively achieved through operational planning and reduction of energy consumption.

Christos et al. [32] proposed an innovative system of secure caching scenario which operates in a wireless-mobile 6G network for managing Big Data (BD) on smart buildings (SBs). The proposed scenario combines the functions of the IoT with Cloud Computing (CC), Edge Computing (EC), and BD (on SB). They created a novel and secure cache decision system (CDS) in a wireless network that operates over an SB, which offered the users a safer and efficient environment for browsing the Internet, sharing and managing large-scale data in the fog.

Christos et al. [33] proposed Integrated Federated Model (InFeMo) to incorporate all the existing cloud models with a federated learning scenario, as well as other related technologies that may have integrated use with each other, offering a novel integrated scenario. The proposed model motivated to deliver a more energy-efficient system architecture and environment for the users. The proposed system was built on the resources made available by Cloud Service Providers (CSPs) and by using the PaaS (Platform as a Service) model, in order to be able to handle user requests better and faster. Their research tried to fill a scientific gap in the field of federated cloud systems.

A few of the recent works on resource allocation in a cloud environment with its solution approach are listed in Table 1.

## 3. Problem Formulation and Network Model

### 3.1. Research Gap

From the review [21,22,23,24,25,26,27,28,29,30,31], many specialists have attempted to accomplish better outcomes in asset allotment, asset arranging, and appropriation. In past reviews, clients did not require assets, and assets were circulated such that clients did not organize. The asset portion is a significant segment of distributed computing. Its exhibition will straightforwardly influence the presentation of the whole cloud climate. Since distributed computing has its qualities, starting asset designation strategies and organization processing calculations do not work in these circumstances. When arranging, the organizer should think about various impediments, including the idea of the undertaking, the size of the errand, the time needed to finish the assignment, the accessibility of assets, the request for the assignment, and the stacking. Assignment arranging is a significant issue in distributed computing. Appropriate arranging of works will prompt proficient utilization of assets.

### 3.2. Research Objectives

- To design and develop dynamic resource allocation and task scheduling process
- To minimize the expected total makespan and maximize throughput through optimal scheduling.

A hybrid machine learning framework addresses performance issues while allocating resources and task scheduling. The proposed technique is simulated using the CloudSim.

### 3.3. Network Model

**Figure 1:**
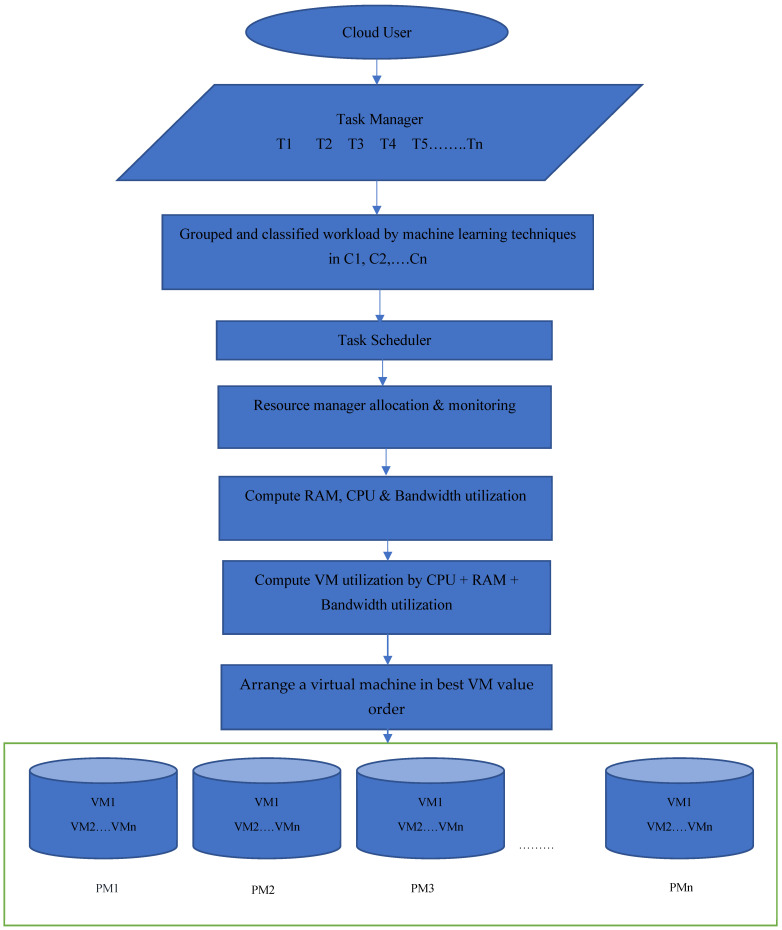
Proposed RATS-HM technique.

The cloud user submits a request to the service provider for access to various resources from the cloud. This requirement is represented as loads. The workload is divided into four categories. The workload is submitted to the task manager and divided into different groups. The work schedule, based on the advanced cat live algorithm, is used to reduce time and increase efficiency. To create a loaded virtual machine (VM), the center value of each cluster is identified and grouped accordingly for the loads. Four clusters are formed: C1, C2, C3, and C4. The detailed model is exhibited in Figure 1. There are a number of Task managers, and the workload is divided into a number of clusters. The ICS-TS algorithm and GO-DNN scheme are used for scheduling tasks and for managing resource allocation. RAM, CPU, and bandwidth utilization of each allocation is computed for each virtual machine and virtual machines are arranged on the best VM value.

## 4. Proposed OEQRM Scheme

This section labels the proposed algorithm for scheduling the workflow. It also explains the three contributions of the proposed algorithm like improved cat swarm optimization algorithm based short scheduler for task scheduling, group optimization-based deep neural network on increasing bandwidth and resource load and lightweight authentication scheme for encrypting the stored data in the cloud. The ICS-TS algorithm in the proposed system accepts some inputs like user request, Task Type, Task Dependency, and Bandwidth and returns multiple service providers to optimize task scheduling. It uses Group Optimized Deep Neural Network (GO-DNN) scheme for optimizing resource utilization. To increase the security of the system, Lightweight, a 64-bit block encryption scheme is used. The following sections present each module of the proposed system.

### 4.1. Task Scheduling with ICS-TS Algorithm

Parameters of multiple service providers for optimizing scheduling are as follows:

**User request (*U_r_*):** Set of user requests which consist of 1 to n task units.

**Task Type (T_t_):** The type of task is described, and it consists of 1 to m. the T_m_ indicates a maximum number of the task inside the task unit.

**Task dependency (*T_d_*):** Dependencies of task units are indicated as *U_r_*. The data obtained from *U_ri_* is given as $T_{d_{\;ij}}=1$ and this data is used by $T_{d_{\;ij}}$. Otherwise, it is considered as $T_{d_{\;ij}}=0$

(1) $$T_{d_{\;33}}=\left[\begin{array}{cccc} & U_{r1} & U_{r2} & U_{r3}\\ U_{r1} & 0 & 1 & 0\\ U_{r2} & 1 & 0 & 0\\ U_{r3} & 0 & 1 & 0 \end{array}\right]$$

**Input Data (D_in_):** The input data size of the task unit is represented as input data.

**Output Data (D_out_):** The yield information size of the errand unit is spoken to as D_out_. For this situation, the asset pool is accepted as heterogeneous, and the asset is taken from the actual machine or a worker or PC in the distance that comprises the information center. Different arrangements are appointed to the same assets. The asset information consists of six tuples, and it is given as R = (PM, P_c_, CP, R, CE, Nbw, Ecom).

**Physical Machines (*PM*):** It indicates the set of actual machines present in a data center, and it is indicated as PM = (PR_1_, PR_2_, PR_3_,..., PR_n_).

**Computing Power (P_c_):** P_c_ indicates the matrix of the processing power of the actual machines. $P_{c_{\;ij}}$ Indicates execution of the task unit type i on a physical machine *PM_j_* and the average power of *PM_j_* is represented by $P_{c\;avg,j}$. The average power is calculated by taking the mean of entries in the column of a matrix $P_{c_{\;j}}$.

(2) $$T_{d_{\;ij}}=\left[\begin{array}{ccccc} & PM_{1} & PM_{2} & \ldots & PM_{j}\\ Ur_{1} & T_{t1,1} & T_{t1,2} & \ldots & T_{t1,j}\\ Ur_{2} & T_{t2,1} & T_{t2,2} & \ldots & T_{t2,j}\\ \vdots & \vdots & \vdots & \vdots & \vdots \\ Ur_{i} & T_{ti,1} & T_{ti,1} & \ldots & T_{ti,j} \end{array}\right]$$

**RAM in PM (R):** Each PM’s existing Random Access Memory.

**Computing Energy (CE):** A matrix indicates a task unit’s execution time. The energy consumed by a *PM_j_* to execute ith task unit type per unit time per unit data is given as CE_ij_.

**Bandwidth (BW):** The bandwidth between PMs and the data transfer rate between *PM_i_* to *PM_j_*, and this is indicated by BW_ij_.

Initially, it is considered that there are k-subtasks for the users in the cloud services. These k-subtasks are scheduled using m computational resources, and it is indicated by {R_1_, R_2_,..., R_m_}. It is considered that each resource, R_j_, has a fixed costs price, and it is indicated as p_j_. The price vector is given by p = {p_1_,p_2_,...,p_m_}.The time allocated for R_i_ to execute the subtask is given t_i_. The execution time vector is given by t = {t_1_,t_2_,….t_m_}.

To enhance the multitude-based bumblebee, a mating calculation is used, and the advancement depends on the pursuit calculation is supported by the way toward mating in genuine bumble bees. The conduct of bumblebees is the collaboration of their:

- ecological and physiological environments,
- genetic potentiality,
- the social conditions of the colony, as well as various earlier and ongoing interactions between these three parameters.

ICS are unique social insects that live in the forces created by them. The three most common elements of the river are the queen, some of the planes, and the workers. The queen is larger than other bees because of the royal jelly. The average lifespan of a queen is five to six years, which can be as high as 1500 eggs. When the queen flies inside the bee, the plane tries to join her in the air. Equation (3) shows a controlled drone crossing with the queen.

(3) $$P(D)=e^{\left[\frac{-\Delta (f)}{S(t)}\right]}$$

where *P*(*D*) indicates the probability of the addition of the queen’s spermatheca to the drone’s sperm (*D*), Δ(*f*) indicates the magnitude of difference between the queen’s spermatheca and the drone’s sperm. The queen’s speed at time ‘t’ is given by *S*(*t*). After iteration, the speed and energy of the queen decrease, and these are represented in Equations (4) and (5), respectively:

(4) S(t+1)=αS(t)

(5) E(t)=αE(t)

where α ranges between [0, 1] is the randomly generated factor. It calculates the decreased energy and speed at each iteration. ‘E’ indicates the energy, and ‘S’ indicates the speed. The detailed algorithm is present below (Algorithm 1).

**Algorithm 1** ICS-TS algorithm
**Input:**

***Din, Ur, Tt, BW***

**Output:**

***Multiple Service Providers To Optimize Scheduling***

**1** 

**Initialize D_in_, U_r_,T_t_, BW**

**2** 

**Calculate the *U_r_* and D_in_ (using it equation)**

**3** 

**Calculate the T_d_.**

**4** 

**Remove the dependent data from the vector.**

**5** 

**Calculate the initial value of *P*(*D*)**

**6** 

**if (*P*(*D*) = = 1)**

**7** 

***U_r_* with higher priority is executed**

**8** 

**Calculate the P_c_,CE**

**9** 

**Keep it best solution**

**10** 

**Else**

**11** 

**Wait until *P*(*D*) become high**

**12** 

**After some iteration if (*P*(*D*) == 0)**

**13** 

**Replace the queen with brood**

**14** 

**End**



**Return: *Multiple Service Providers To Optimize Scheduling***


The complexity of the ICS-TS algorithm algorithm(Algorithm 1) is O(n) where n is number of times the value of *P*(*D*) is non-zero.

### 4.2. Resource Allocation Using GO-DNN

The important goal is to optimize the allocation of resources. The use of resources creates a layer to increase the efficiency of cloud systems. Reducing the cost of using existing resources is another factor.

•→ makespan = min ((F_ji_) for j_i_ ∈ J

•→ Cost = min(C (r_i_, j_k_)) for 1 ≤ I ≤ a, 1 ≤ k ≤ b (6)

•→ Fitness = αCost + βmakespan + βreliabilty

where α, β, and γ are in [0, 1]. They are parameters to influence the variables of fitness. C(r_i_, j_k_) is the cost of the job j_k,_ which executes on resource r_j,_ and makespan is the termination time of the job. Similarly, if the movement of bacterium varies for a period of time, it is tumbling. The p^th^ bacterium atq^th^ chemotactic r^th^ reproductive and t^th^ elimination and dispersal step is given by *α^p^*(*q*, *r*, *t*). The step size of the tumble is given by *S*(*p*). The computation chemotaxis is described as:

(7) $$\alpha^{p}(q+1,r,t)=\alpha^{p}(q,r,t)+S(p)$$

The parameter used in the BEA algorithm is d—dimension of the search space, N—number of bacteria in the *N*_c_—chemotaxis step, *N*_s_—swim a length, *N*_r__e_—the number of reproduction steps, *N*_e__d_—the number of elimination dispersal events, *P*_e__d_—elimination-dispersal with probability, S (i)—the size of the step taken in the random direction.

Only optimal policies are considered; any reference to an optimal policy in the sequel pertains to an optimal policy or value function for the clairvoyant problem.

(8) $$CH_{i}=\sum_{i=1}^{n}S_{A_{i}}$$

Finally, the Brown function [9] is used to crosscheck the resource allocation as follows:

(9) $$F\left(S_{A}\right)=\sum_{i=1}^{n}{\left(S_{A_{i}}^{2}\right)}^{\left(S_{A_{i+1}}^{2}+1\right)}+{\left(S_{A_{i+1}}^{2}\right)}^{\left(S_{A_{i}}^{2}+1\right)}$$

Generally, when multiple sensors are spread over a locality, each node is configured with equal sensing equipment. Hence any failure of one or more nodes can be handled by the network without much inconvenience. Thus, to evaluate the time efficiency of the virtual machine, the time elapsed until the first node died cannot be taken as the only metric.

(10) $$t_{i}=\sum_{j=1}^{\left[p_{i}\right]}\lambda^{p_{1}}\frac{x^{p_{1}-1}e^{-\lambda \tau }}{\Gamma \left(p_{i}\right)}$$

where $p_{i}$ is the upper bound on a number of the virtual machine that can be transmitted by sensor *i* during the time τ.

(11) $$NLT=T\left[\max\left(t_{i}\right)\in \frac{N_{a}}{N}\right]$$

where *N_a_* holds the number of nodes alive and *N* holds the number of sensors in the network. The cost of routing(*CR*) between two nodes are presented in (12),

(12) $$CR(nd)=\sum_{i,j\in (n,\cup ,d)}C_{i,j}$$

where *C_i,j_* is the cost function for a connection from node *i* to node *j*. Thus,

(13) $$C_{i,j}=E_{p}+2NE_{tx}\left(n,d\right)+e^{\frac{1}{E_{R}^{i}}}$$

where, $E_{R}^{i}$ is a cost function that acts as balancing factors for sensors’ energy consumption, considering the remaining energy of sensors.

The velocity of an agent is calculated by adding its acceleration (Equation (15)) to the fraction of its current velocity (Equation (16)).

The next location of the agent can be calculated using Equation (17):

(14) $$F_{i}^{d}(t)=\sum_{j\in kbest,j\neq i}rnd_{j}G(t)\frac{M_{j}(t)M_{i}(t)}{R_{ij}+\varepsilon }\left(x_{j}^{d}(t)-x_{i}^{d}(t)\right)$$

(15) $$a_{i}^{d}(t)=\frac{F_{i}^{d}(t)}{M_{i}(t)}=\sum_{j\in kbest,j\neq i}rnd_{j}G(t)\frac{M_{j}(t)}{R_{ij}+\varepsilon }\left(x_{j}^{d}(t)-x_{i}^{d}(t)\right)$$

(16) $$V_{i}^{d}(t+1)=rnd_{i}\times V_{i}^{d}(t)+a_{i}^{d}(t)$$

(17) $$X_{i}^{d}(t+1)=X_{i}^{d}(t)+V_{i}^{d}(t+1)$$

where *rnd_i_* and *rnd_j_* are random numbers in the interval [0, 1], *ε* is a small value, R_ij_(t) indicates the Euclidean distance from agent I to agent *j* and is calculated ||X_i_(t) − X_j_(t)||^2^. K_best_ is the set of first K agents having the best fitness value. K is a function of time initialized to K_initial_ value, which will be reduced time.

The gravitational constant is represented by *U*(*t*) and holds the initial value as *U_initial_*:

(18) $$U(t)=U(U_{initial},U_{end},t)$$

K and U are two main components used for balancing its diversification and intensification in GSA. Diversification is used to prevent being trapped in the local optimum at initial iterations.The detaild algorithm is present below (Algorithm 2).

**Algorithm 2** Multi-objective Rule Set 
**Input:**

***d*, *N*, *Nc*, *Ns*, *Nre*, *Ned*, *Ped*, *S* (*i*)**

**Output:**

***Optimal Resource utilization***

**1** 

**Initialization of the parameters: d, N,** ***N*c,** ***N*s,** ***N*re,** ***N*ed,** ***P*ed,S(i)**

**2** 

**Elimination-dispersal loop is taken by k = k + 1**

**3** 

**Reproduction loop is represented by: l = l + 1**

**4** 

**Chemo taxis loop is given by m = m + 1**

**5** 

**Select the heuristic.**

**6** 

**Calculate fitness function F (p, m,l,k) and it is given by**

**7** 

**F(p, m, l, k) = J(p, m, l, k) + Fcc(** **αP(m, l, k),L(m, l, k))**

**8** 

**Assume Flast = F (p, m, l, k)**

**9** 

**Tumble: create a random vector Δ (i)** **∈Rn with each ∆j(p), j = 1, 2, 3……d.**

**10** 

**Move:** **αP(m + 1,l,k) =** **αP(m, l, k)+ S(P)(Δ(P)/ √∆** **T(P)Δ(P))**

**11** 

**Calculate F(p, m, l, k) and F(p, m, l, k)+ Fcc(** **αP(m, l,k),P(m, l, k))**

**12** 

**Swim**

**13** 

**Assume j = 0.**

**14** 

**While j<** **Ns, j = j + 1**

**15** 

**if F(p, m + 1, l, k) < Flast, let Flast = F(p,m + 1,k,l) and** **αP(m + 1,l,k)=** **αP(m,l,k) + S(P)(Δ(P)/ √∆** **T(P)Δ(P)) and use this** **αP(m + 1,l,k) to calculate the new F(p,m + 1,l,k)**

**16** 

**else let j =** **Ns**

**17** 

**End**

**18** 

**Iterate to next bacteria (p + 1) if p ≠ N.**

**19** 

**End**




**Return: *Optimized Resource Utilization***



The addition of high values to K and G parameters in the initial stage is considered an important step in the GSA, and it is indicated as K_initial_ and *U_initial_*. If high-value K is used, the mass will be moved to the search space based on the position of more masses, thereby increasing the diversification of the algorithm. High-value G is used to increase the mobility of each mass present in the search space, thereby increasing the diversification of the algorithm. The best solution space can be identified by assuming high values of K and G. The complexity of the above algorithm is O(***Ns*** × ***N***). This is a swarm-based algorithm where the execution depends on the value of ***Ns*** and ***N***.

### 4.3. Data Encryption Using Lightweight Scheme

Lightweight is an encryption algorithm based on block cipher for cloud computing and is suitable for constraint-resource applications. Lightweight uses a text of 64-bit block length and a key of 128 bit long. It uses a Feistel network structure and it comprises of 32-rounds. Three different lightweight operations are left bit-wise rotation, addition mod 28, and XOR. The following notations are used to describe lightweight.

The 64-bit plaintext and ciphertext are considered concatenations of 8 bytes and denoted by $T=T_{7}//T_{6}//\ldots \ldots \ldots //T_{0}$ and $F=F_{7}//F_{6}//\ldots \ldots //F_{0}$ respectively. Similarly the 64-bit intermediate values are represented as, $Y_{i}=Y_{i,7}//Y_{i,6}//\ldots \ldots //Y_{i,0}$ for *i* = 0, 1, 2,…,32.

The lightweight uses a 128-bit master key, a concatenation of 16 bytes and denoted by MK = MK_15_||……||MK_0_. The followings are notations for mathematical operations:

The encryption process of plain text T

(19) $$T=T_{7}//T_{6}//T_{5}//T_{4}//T_{3}//T_{2}//T_{1}//T_{0}$$

#### 4.3.1. Initial Transformation

Initial Transformation transforms a plaintext T given as input to the first Round Function, $Y_{0}=Y_{0,7}//Y_{0,6}//\ldots \ldots //Y_{0,0}$ by using the four whitening-key bytes, *WK*_0,_ *WK*_1,_ *WK*_2_ and *Wk*_3_

(20) $$Y_{0,0}=T_{0}⊕WK_{0,}\;\;X_{0,1}=T_{1,}$$

(21) $$Y_{0,2}=T_{2}⊕WK_{1,\;\;\;}Y_{0,3}=T_{3,}$$

(22) $$Y_{0,4}=T_{4}⊕WK_{2,\;\;\;}Y_{0,5}=T_{5,}$$

(23) $$Y_{0,6}=T_{6}⊕WK_{3,\;\;\;}Y_{0,7}=T_{7,}$$

For *i* = 0 to 30

(24) $$Y_{i+1,0}=Y_{i,7}⊕(F_{0}(Y_{i,6})⊕SK_{4i+3}),Y_{i+1,1}=Y_{i,0,}$$

(25) $$Y_{i+1,2}=Y_{i,1}⊕(F_{1}(Y_{i,0})⊕SK_{4i}),Y_{i+1,3}=Y_{i,2,}$$

(26) $$Y_{i+1,4}=Y_{i,3}⊕(F_{0}(Y_{i,2})⊕K_{4i+1}),Y_{i+1,5}=Y_{i,4,}$$

(27) $$Y_{i+1,6}=Y_{i,5}⊕(F_{1}(Y_{i,4})⊕SK_{4i+2}),Y_{i+1,7}=Y_{i,6,}$$

#### 4.3.2. Final Transformation

Final Transformation untwists the swap of the last round function and transforms $Y_{32}=Y_{32,7}//Y_{32,6}//\ldots \ldots //Y_{32,0}$ it to the ciphertext F by using the four whitening-key bytes *WK*_4,_ *WK*_5,_ *WK*_6, and_ *Wk*_7._ This step is similar to the initial Transformation. It is observed that the X-OR and modular arithmetic operations are performed to generate the seven-byte ciphertext.

(28) $$F_{0}=Y_{32,0}⊕WK_{4,}\;F_{1}=Y_{32,1,}$$

(29) $$F_{2}=Y_{32,0}⊕WK_{5,}\;F_{3}=Y_{32,3,}$$

(30) $$F_{4}=Y_{32,4}⊕WK_{6,}\;F_{5}=Y_{32,5,}$$

(31) $$F_{6}=Y_{32,6}⊕WK_{7,}\;F_{7}=Y_{32,7,}$$

$F=F_{7}//F_{6}//F_{5}//F_{4}//F_{3}//F_{2}//F_{1}//F_{0}$ (*F_i_* are ciphertext bytes)

The decryption operation is identical in operation to encryption apart from the following modifications. Operations replace all operations except for the operations connecting and output of *F*_0_. The order in which the keys *WK_i_* and *SK_i_* are applied is reversed.

## 5. Results and Discussion

The proposed model is simulated using a cloudlet simulator, and test results are evaluated to measure its performance. Based on the obtained results, some factors are like resource utilization, acquisition speed, implementation time, and energy management are analyzed. Create a cloud data center measured in continuous PM. It also starts creating data canters with resource agents. Each data center started with multiple data hosts and associated VMs. Client Tasks Cloudlets and Cloudlet Planning address incoming tasks. We compared our proposed RATS-HM task planning, an optimal power minimization (ITSEPM), First Coming First Serve (FCFS), and Round Robin (RR). Table 2 and Table 3 show the hardware and simulation settings.

The proposed (RATS-HM) system improves performance and integrates with some existing methods. We evaluate the performance of our proposed model using different parameters such as power consumption, data center resource usage, acceptance rate, and implementation time. Resource usage is calculated as the ratio of data center resources, CPU, memory, bandwidth, and total capacity. We use the block processing concept for Visit official visit technology. At the same time, jobs come in t = 0.

For distribution, we use a specific distribution system. The work planning concept used in our framework prioritizes tasks. Priority is given to agents who allocate resources from the resource table. Process acquisition speed, velocity, and execution time are calculated, and standard scheduling algorithms analyze FCFS and round-robin methods.

### 5.1. Performance Metrics

Metrics like power consumption, resource utilization, bandwidth utilization, memory utilization, and response time are used to evaluate the proposed model and its comparison with some of the existing ones.

#### 5.1.1. Evaluation of Resource Utilization

It refers to the number of allocated resources a task spends for its complete execution. Resource Utilization (*R*_*U*_) can be represented as

$$R_{U}=R_{avl}-R_{nu}$$

where *R_avl_* denotes resources available and *R_nu_* denotes unutilized resources.

**Figure 2:**
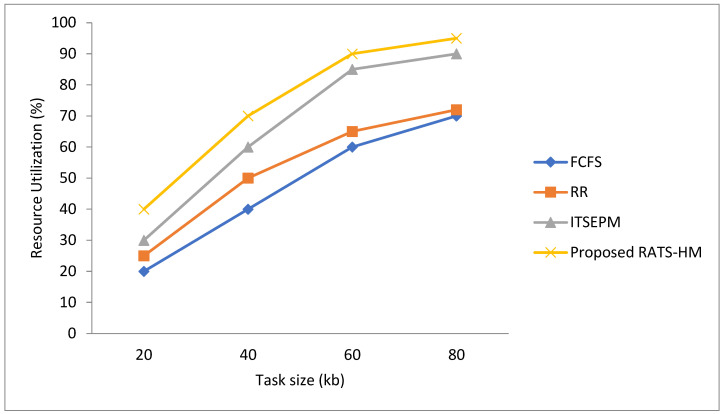
Resource utilization with proposed and existing techniques.

Resource usage includes our specific work processor and memory usage. The percentage of using a particular method is always higher when the other two methods are combined. Figure 2 shows a graphic representation of the percentage utilization of resources by using various resource allocation schemes. It shows that for various task sizes, the percentage utilization of resources is maximum for the proposed RATS-HM technique.

#### 5.1.2. Evaluation of Response Time

Response time of a task is the time elapsed between the launching of a task and its completion of execution. The response time *TS*_Re*s*_ can be represented as follows,

TSRes=TSCT−TSAT

where *TS_CT_* is the completion time of the task and *TS_AT_* is the arrival time of the task. The response time is presented in Table 4.

**Figure 3:**
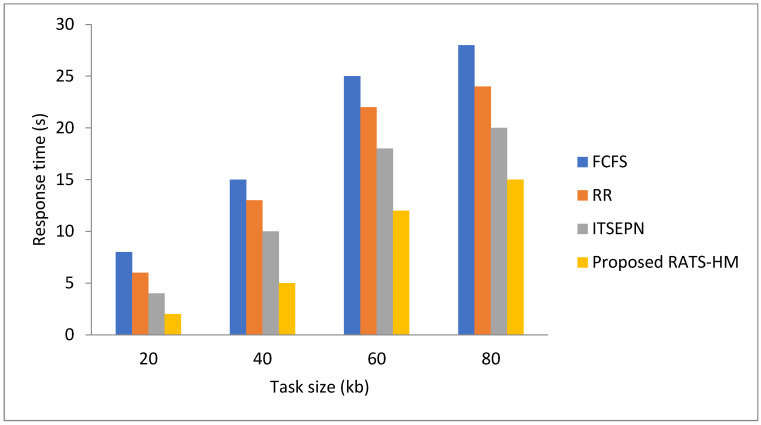
Comparison of responses time.

Resource usage includes our specific work processor and memory usage. The percentage of using a particular method is always higher when the other two methods are combined. Figure 3 shows the maximum resource usage. This is essentially the RATS-HM proposed in our work in which passive prime ministers should be turned off. It participates in the resource utilization system.

#### 5.1.3. Evaluation of Power Consumption

It can be defined as the unit of energy that all cloud servers use in allocating resources. In this specific task, management implements an energy management module to reduce energy consumption. While real-time data centers use many power consumption technologies like dynamic voltage, frequency, and resource sleep, they are not enough for the virtualized environment.

**Figure 4:**
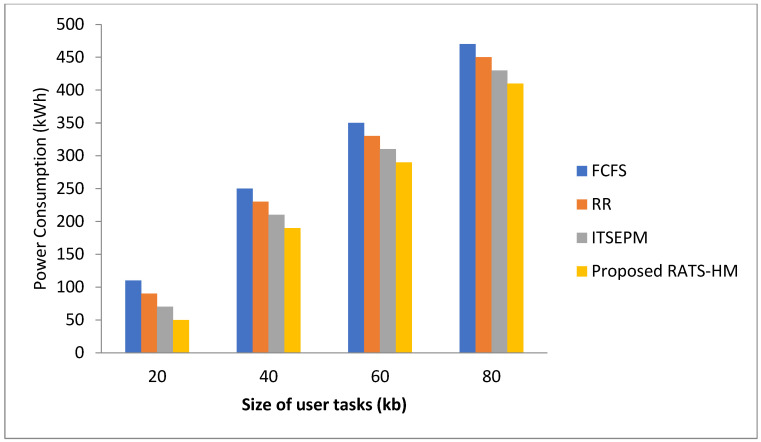
Existing versus proposed RATS-HM technique in terms of power consumption.

Compared to existing ones, our proposed approach gives better results showing energy reduction. Figure 4 is the proof of this. The energy management technique presented in this study reduces passive energy consumption, external energy consumption, internal communication, and primary energy consumption PM.

The appropriate use of assets produces benefits for distributed computing specialist organizations. The exploratory outcome shows that the proposed technique used the CPU asset more proficiently than the current ITSEPM structure. The experimental result shows that the proposed RATS-HM strategy uses memory resources more proficiently than the current FCFS, ITSEPM, and round-robin (RR) systems.

## 6. Conclusions

In this work, we presented a hybrid machine learning algorithm that schedules tasks and efficiently allocates resources in cloud environments. We utilized improved feline multitude advancement calculation, bunch streamlining the based profound neural organization, and a lightweight confirmation plan to expand the memory, CPU, asset, and data transmission. We discovered that our methodology delivers favorable outcomes when we contrast our proposed RATS-HM strategy and the current ITSEPM, FCFS, and Round robin systems for CPU usage and reaction time. Furthermore, from asset use, the proposed RATS-HM method effectively designates assets with high utility. We obtained the most extreme usage result for processing assets, e.g., CPU, memory, and data transfer capacity. The proposed framework adds transmission capacity to two memory and CPU assets. Next, work will zero in on more viable processing to improve utilization time. In the future, a large amount of practical data with a real cloud environment will be used to establish the effectiveness of the proposed model in a real-life scenario.

## Figures and Tables

**Table 1:** Summary of Related Works on Resource allocation in Cloud computing and their proposed solutions.

Citation	Author	Title	Propose Solutions	Environment	Open Issue
[21]	Wei et al.	Imperfect information dynamic Stackelberg game based resource allocation using hidden Markov for cloud computing	The assessed cost of CSAMIISG is near the genuine exchange cost and the exchange cost is not exactly the real exchange esteem	Huawei	Application framework and change settings to make it more effective
[22]	Tang et al.	Fair resource allocation for data-intensive computing in the cloud	The technique offers various leveled long haul asset reasonableness (H-LTRF) with the option of the LTRF expansion to add progressive sources, for example, the LTRF and H-LTRF.	Amazon EC2	LTYARN open source at
					http://sourceforge.net/projects/ltyarn/ (accessed on 22 August 2021)
[23]	Zhang et al.	An online auction mechanism for cloud computing resource	The author proposes the online virtual resource allocation and payment (OVRAP) algorithm	IBM CPLEX12	C++ is used for algorithm implementation
		allocation and pricing based on user evaluation and cost			
[24]	Jiang et al.	Self-adaptive resource allocation for energy-aware virtual machine placement in a dynamic computing cloud	proposed method first groups the servers with a shorter path length using the given DCN topology	Google cluster trace	Lacks a large amount of practical data
[26]	Wu et al.	ANFIS with natural language processing and gray relational analysis based cloud computing framework for real-time energy-efficient resource allocation	proposed aANFIS model solves the dynamical prediction problem of VM workload by training the values of feature attributes	Malleable Network System Simulator	Lacks a large amount of practical data

**Table 2:** Hardware Specifications.

Required	Component Specification
Processor	Intel^®^ Pentium^®^ CPU G2030 @ 3.00 GHZ
Operating System	Windows (X86 ultimate) 64-bit OS
Hard Disk	1 TB
RAM	4 GB
System	64 Bit OS System

**Table 3:** Simulation Settings.

Component	Specification	Values
Cloudlets	Length of task No of tasks	1600–3400 30–300
Virtual Machine	Host	4
Physical Machine	Memory Bandwidth Storage	540 25,00,00 500 GB

**Table 4:** Evaluation and analysis of response time.

Offline	Execution Time
Workload prediction online	10 min
Task monitoring and scheduling	20 min
Connection to agents	0.050 s
Power management	2.015 s
Response to users	0.010 s
